# Neuraminidase-3 Is a Negative Regulator of LFA-1 Adhesion

**DOI:** 10.3389/fchem.2019.00791

**Published:** 2019-11-22

**Authors:** Md. Amran Howlader, Caishun Li, Chunxia Zou, Radhika Chakraberty, Njuacha Ebesoh, Christopher W. Cairo

**Affiliations:** Department of Chemistry, University of Alberta, Edmonton, AB, Canada

**Keywords:** integrin, adhesion, glycolipid, glycosyl hydrolase, inflammation

## Abstract

Within the plasma membrane environment, glycoconjugate-receptor interactions play an important role in the regulation of cell-cell interactions. We have investigated the mechanism and activity of the human neuraminidase (NEU) isoenzyme, NEU3, on T cell adhesion receptors. The enzyme is known to prefer glycolipid substrates, and we confirmed that exogenous enzyme altered the glycolipid composition of cells. NEU3 was able to modify the sialic acid content of purified LFA-1 *in vitro*. Enzymatic activity of NEU3 resulted in re-organization of LFA-1 into large clusters on the membrane. This change was facilitated by an increase in the lateral mobility of LFA-1 upon NEU3 treatment. Changes to the lateral mobility of LFA-1 were specific for NEU3 activity, and we observed no significant change in diffusion when cells were treated with a bacterial NEU (NanI). Furthermore, we found that NEU3 treatment of cells increased surface expression levels of LFA-1. We observed that NEU3-treated cells had suppressed LFA-1 adhesion to an ICAM-1 coated surface using an *in vitro* static adhesion assay. These results establish that NEU3 can modulate glycoconjugate composition and contribute to the regulation of integrin activity. We propose that NEU3 should be investigated to determine its role on LFA-1 within the inflammatory cascade.

## Introduction

The process of leukocyte rolling, extravasation, and homing to sites of inflammation is critical to cellular immunity, and is known as the leukocyte adhesion cascade (Ley et al., 2007). Along each step of this process different cell adhesion molecules and their ligands mediate recognition between leukocytes and endothelial cells. The initial attachment of the leukocyte to the endothelial wall, usually referred to as rolling, is mediated by selectins and their carbohydrate ligands (e.g., sialyl Lewis-X; CD15s) (Varki, 1994). Later steps of the process must arrest the cell (firm adhesion) to allow for transmigration. These later steps of the process are largely mediated by integrin receptors and their ligands. Integrins are a major class of adhesion receptors and an important therapeutic target (Hynes, 2002; Cox et al., 2010; Desgrosellier and Cheresh, 2010). The first integrin in the inflammatory cascade is LFA-1 (known as the αLβ2 integrin; or CD11a, CD18), a transmembrane glycoprotein which binds to ICAM-1 (inter-cellular adhesion molecule-1; CD54) and conveys an outside-in intracellular signal to the leukocyte (Hogg et al., 2004). These and subsequent integrin-mediated processes, including interactions of the very-late antigens (VLA-4, the α4β1 integrin or VLA-5, the α5β1 integrin), allow cells to migrate to the site of inflammation (Hogg et al., 2003; Simmons, 2005; Cox et al., 2010). Thus, processes which modulate leukocyte integrin function are of potential interest for the development of anti-adhesive and anti-inflammatory therapeutics (Hogg et al., 2003; Simmons, 2005; Cox et al., 2010).

Cell surface glycoconjugates are critical components of the plasma membrane. Sialic acid-containing glycolipids, known as gangliosides, play important structural and functional roles. Sialic acid (also known as neuraminic acid, or Neu5Ac) has long been recognized to participate in the regulation of immune cell function. The sialic acid content of lymphocyte receptors is known to be altered as part of cell development (Bi and Baum, 2009), infection (Galvan et al., 1998), and activation (Hernandez et al., 2007). The enzymes that remove sialic acid, known as neuraminidases (NEU; also called sialidases), increase trans-endothelial migration (Sakarya et al., 2004), reduce expression of CD15s (Gadhoum and Sackstein, 2008), and expose integrin activation epitopes (Feng et al., 2011). Early reports dubbed increases in B cell antigen sensitivity a “neuraminidase effect,” (Cowing and Chapdelaine, 1983) and recent evidence has ascribed this phenomenon to sialic acid acting as a negative regulator of immune cell interactions (Bagriacik and Miller, 1999). The prominent role of sialic acid in adhesion suggests that changes which affect sialoglycoconjugates (SGC) may be critical to regulation of cell-cell interactions.

Catabolic remodeling of glycoconjugates is likely to be more rapid than biosynthetic processes (Parker and Kohler, 2010). Membrane-associated glycosyl hydrolase (GH) enzymes could play a role in signaling pathways through processing of glycolipids or glycoproteins. This hypothesis is consistent with the increased turnover of terminal glycan residues (e.g., neuraminic acid and fucose) relative to core glycan residues (Tauber et al., 1983), and the rapid loss of sialylated antigens on neutrophils (Gadhoum and Sackstein, 2008). The family of human neuraminidases (hNEU) have been shown to participate in a variety of signaling pathways and pathologies including inflammation, adhesion, tumorigenesis, and cancer metastasis (Miyagi, 2010; Miyagi and Yamaguchi, 2012). However, the role of specific hNEU isoenzymes has not been well-defined within inflammation.

The NEU3 isoenzyme is known as a plasma-membrane-associated GH which has a strong preference for glycolipid targets (Monti et al., 2000; Kopitz et al., 2001; Papini et al., 2004; Seyrantepe et al., 2004; Zanchetti et al., 2007). Interestingly, NEU3 has been shown to modulate β1 integrin activity (Tringali et al., 2012). Additionally, the enzymatic activity of NEU3 is modulated by signaling events such as protein kinase C stimulation in immune cells (Wang et al., 2004). The specificity of NEU3 for glycolipids, and its localization to membrane microdomains (Wang Y. et al., 2002), suggests a central role for the enzyme in cellular signaling (Kopitz et al., 2001). The glycolipid GM3 is a key component of lipid rafts, as well as a substrate for NEU3 (Sandbhor et al., 2011). Our group has been interested in the function of NEU3 in regulating membrane organization. We wanted to investigate the effects of NEU3 on integrin-mediated leukocyte adhesion through its regulation of SGC. Glycolipid interactions with integrins have been examined by a number of groups (Pande, 2000). Lactosyl ceramide (LacCer) has been shown to activate β1 integrins (Sharma et al., 2005; Chatterjee and Pandey, 2008). The activation of LFA-1 in neutrophils has been found to require LacCer-enriched domains (Chatterjee and Pandey, 2008). Imaging studies have found that LFA-1 on monocytes is associated with raft markers (Cambi et al., 2006); and activation of cells allows LFA-1 nanodomains to assemble into larger clusters with GPI-associated proteins (van Zanten et al., 2009). Taken together, these reports suggest an important role for glycolipids in the regulation of integrin organization and function on lymphocytes.

In this study, we investigated the role of the human NEU3 isoenzyme in regulating LFA-1 adhesion in a T cell model (Jurkat) and peripheral blood mononuclear cells (PBMC). We found that exogenous enzyme altered the glycolipid composition of cells, as well as the organization of LFA-1 in the membrane. By measuring the lateral mobility of LFA-1, we provide mechanistic insight into the altered distribution of LFA-1. We observed that NEU3 activity significantly increased LFA-1 lateral mobility and endocytosis, and blocked LFA-1–ICAM-1 adhesion. We also found that NEU3 treatment did not block all adhesion pathways, as homotypic aggregation of cells was increased. Together, our results suggest that NEU3 may have a role in the regulation of lymphocyte integrins critical to the inflammatory cascade.

## Results

### NEU3 Treatment Reduced Sialylated-Glycolipids in Cells

**Figure 1:**
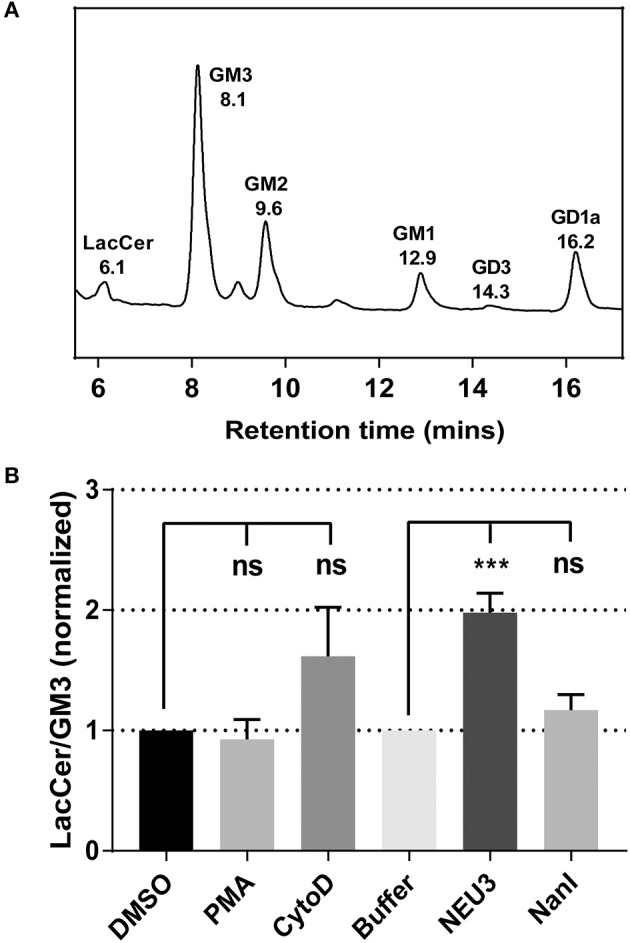
Analysis of the change of cell membrane GSLs. GSLs were extracted from treated or control cells and analyzed by LC-MS. **(A)** Glycolipids extracted from Jurkat cells were digested with endoglycoceramidase, labeled and resolved by LC-MS-FLD. The major glycolipids observed were LacCer, GM3, GM2, GM1, and GD1a. **(B)** LC-MS-FLD analysis was performed on four replicate samples (*N* = 4) for Jurkat cells treated as indicated. The ratio of LacCer to GM3 was calculated using the peak areas for each condition and normalized to the respective control. Data were compared to the indicated control using a student *t*-test to determine *p*-values; ***p ≤ 0.005; ns, not significant.

To gain insight into gross changes in the composition of membrane glycosphinolipids (GSL), we first employed high-performance thin layer chromatography (HPTLC). Jurkat T cells were treated with conditions expected to alter integrin function, and GSL were extracted and analyzed by HPTLC (Muthing, 1996). We observed only minor variations in sialo- and asialo-forms of gangliosides which were difficult to quantitate (data not shown). To provide more quantitative insights we implemented an LC-MS-FLD analysis of gangliosides based on previous reports (Neville et al., 2004; Albrecht et al., 2016). We detected glycolipids extracted from Jurkat cells including LacCer, GM1, GM2, GM3, and GD1a (Figure 1A and Figure S7). We focused on changes to the ratio of LacCer to GM3 since GM3 is a well-known substrate for NEU3. This analysis showed no significant changes on treatment with phorbol 12-myristate 13-acetate (PMA; a protein kinase C activator), and minor, but not significant, changes with cytochalasin D (cytoD; a cytoskeletal disruptor) (van Kooyk and Figdor, 2000). Human cell types typically express multiple isoforms of NEU (Miyagi and Yamaguchi, 2012). In order to probe the role of a single NEU isoenzyme in cells, we treated cells with recombinant NEU3 enzyme and a bacterial NEU from *Clostridium perfringens* (NanI) (Peter et al., 1995; Albohy et al., 2010). We found that treatment with NanI had no detectable effect on glycolipid composition; however, NEU3 showed a significant increase in asialo forms of GM3 (Figure 1B). This result suggested that NanI did not substantially alter ganglioside composition, while NEU3 showed more specific activity for glycolipid substrates (Ha et al., 2004; Sandbhor et al., 2011). We concluded that treatment of cells with NEU3 resulted in an altered composition of membrane glycolipids, which included reduction in GM3 and an increase in LacCer.

### NEU3 Treatment Altered the Glycosylation of LFA-1

**Figure 2:**
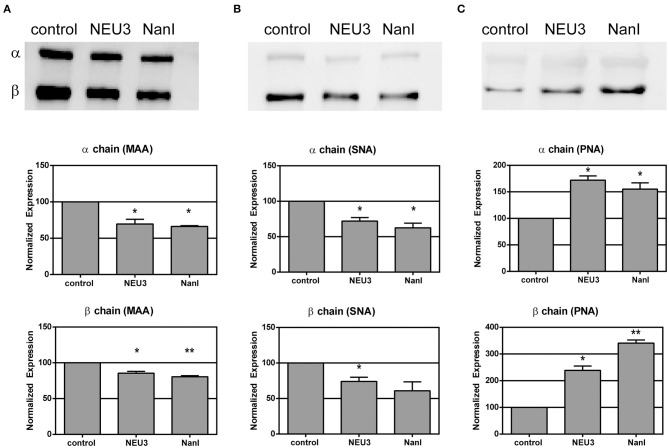
Lectin blotting of LFA-1 shows sensitivity of LFA-1 to NEU treatment. Purified LFA-1 was treated with NEU3 and NanI for 3 h at 37°C. The protein was then blotted and probed with biotinylated lectins. Lectins **(A)** MAA, **(B)** SNA, and **(C)** PNA were used. MAA and SNA recognize terminal sialic acid residues, while PNA recognizes terminal galactose residues. Chemiluminescent blots were developed and analyzed for changes in band intensities, and a representative image from two experiments are shown at the top of each panel (see Supporting Information). Data are shown as the mean ± SEM and were compared to the appropriate control using *t*-test to determine *p*-values; *p ≤ 0.05; **p ≤ 0.01.

We used lectin blotting to detect changes in the glycosylation state of LFA-1 after NEU treatment (Figure 2 and Figures S4–S6). We selected the *Sambucus nigra* agglutinin (SNA), peanut agglutinin (PNA), and *Maackia amurensis* agglutinin (MAA) for this analysis. The PNA lectin binds terminal galactose residues, while SNA and MAA bind to terminal sialic acid residues (Freeze, 2001). We observed that treatment of purified LFA-1 with NEU3 and NanI resulted in a significant decrease in SNA and MAA staining for LFA-1, consistent with loss of sialic acid. Treatment with either NEU enzyme gave a corresponding increase in PNA staining, suggesting a corresponding increase in terminal galactose residues after loss of sialic acid. These results were consistent for both the α- and β-chains of LFA-1. Together, these data are consistent with desialylation of the LFA-1 complex, leading to an increased amount of exposed galactose sites in the presence of NEU3 or NanI activity.

### Fluorescence Imaging of LFA-1

**Figure 3:**
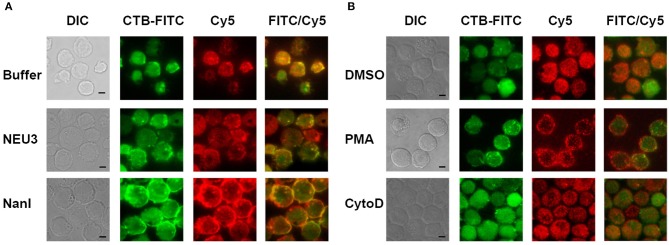
TIRF imaging of LFA-1 on treated cells. Jurkat cells were fixed after treatment with **(A)** buffer (control), NEU3, or *NanI*; and **(B)** DMSO, PMA, or cytoD. The fixed cells were then labeled with Cholera toxin subunit B (CTB-FITC) to label gangliosides and a TS2/4-Cy5 conjugate to label LFA-1. Cells were imaged by DIC and TIRF. Merged FITC and Cy5 images are shown in the last column with yellow indicating co-localization. Scale Bar = 5 μm.

We next sought to determine if NEU3 treatment of cells would result in changes to the localization of LFA-1. Cells were imaged by total internal reflection fluorescence (TIRF) microscopy, limiting visualization to portions of the cell in close apposition to the glass surface. Cells were stained with a Cy5-conjugated anti-LFA-1 antibody (clone TS2/4) and a FITC-conjugated Cholera Toxin subunit B (CTB-FITC) to visualize gangliosides (Blank et al., 2007). Untreated cells showed relatively diffuse LFA-1 microclusters, while CTB gave diffuse staining and large patches with partial LFA-1–CTB colocalization (Figure 3A). Treatment of cells with NEU3 resulted in more punctate CTB staining and more diffuse LFA-1 microclusters. In contrast, NanI treatment resulted in larger co-localized regions of LFA-1 and CTB staining. A portion of the localized aggregates appeared at cell-cell contacts. Treatment of cells with PMA resulted in larger and more distinct microclusters of LFA-1 and minimal CTB colocalization (Figure 3B). Treatment of cells with cytoD disrupted CTB-positive aggregates and reduced co-localization with LFA-1 microclusters. LFA-1 is known to form nanoclusters on resting and activated cells, and the membrane domains in which LFA-1 is found tend to be heterogeneous (Marwali et al., 2003; Cambi et al., 2006). We also note that CTB staining may include reactivity to glycoprotein antigens, and therefore imaging results with this stain should be interpreted with caution. Previous reports have suggested that GM1 is the major CTB reactive glycoconjugate in Jurkat cells (Wands et al., 2015).

**Figure 4:**
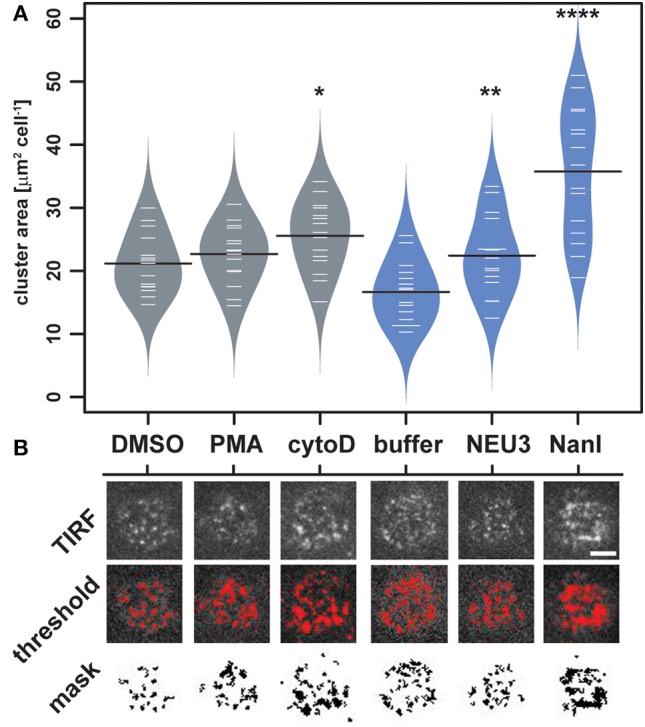
Changes in LFA-1 clustering after neuraminidase treatment. The amount of clustered LFA-1 on individual cells was determined using TIRF imaging. Cells were labeled with a TS2/4-Cy5 antibody conjugate, and 15 individual cells were compared for each condition. **(A)** Clusters that were larger than 4 pixel^2^ were measured using ImageJ, and the average area was tabulated for each cell. Data are plotted using a beanplot, with solid lines representing the mean of each population (Kampstra, 2008). **(B)** Representative TIRF images for individual cells are shown from each condition. A mask was generated from the TIRF image using a threshold for quantitation. Data were compared to the appropriate control using a *t*-test to determine *p*-values; *p ≤ 0.05; **p ≤ 0.01; ****p ≤ 0.0001. Scale bar = 5 μm.

To quantitate changes in LFA-1 cluster size, we analyzed TIRF images of individual cells (*N* = 15) from each condition by determining the amount of LFA-1 found in clusters. Images were processed in ImageJ to identify clusters and to determine the total area per cell found within them (Figure 4 and Table S2) (Schneider et al., 2012). The distribution of total cluster area per cell is shown in Figure 4A. Clear increases in cluster size were observed for cytoD, NEU3, and NanI treatments. Treatment with NanI showed the largest increase in cluster area (consistent with Figure 3). Our observation that NEU3 has similar effects to cytoD in both lateral mobility and clustering indicated that enzyme activity influenced cytoskeletal regulation of the receptor (Cairo et al., 2006; Cairo and Golan, 2008).

### Lateral Mobility of LFA-1 Was Altered by NEU3 Treatment

**Figure 5:**
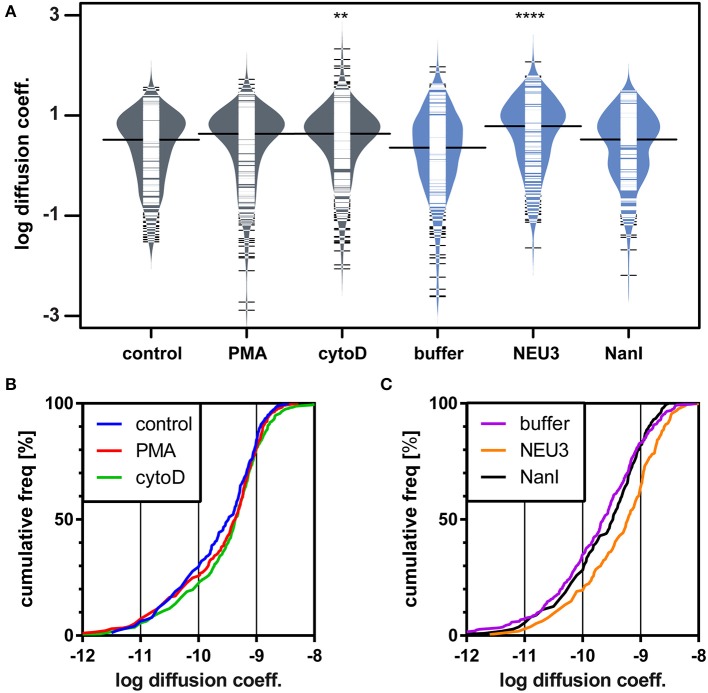
LFA-1 diffusion is altered by NEU treatment. The lateral mobility of LFA-1 on Jurkat was determined using SPT. **(A)** A beanplot of each population is shown with the logarithmic median of diffusion coefficients indicated by a solid line for each population (same data as in Table 1) (Kampstra, 2008). Each population is shown with a density estimate and horizontal lines indicate individual diffusion coefficient measurements. **(B)** A cumulative frequency distribution is shown for the control, PMA, and cytoD conditions. **(C)** A cumulative frequency distribution is shown for the buffer, NEU3, and NanI conditions. Diffusion coefficients are given as log(D), where D is in units of × 10^−10^ [cm^2^s^−1^] or × 10^−2^ [μm^2^s^−1^]. Data were compared to the appropriate control using a *t*-test to determine *p*-values; **p ≤ 0.01; ****p ≤ 0.0001.

We next examined the lateral mobility of LFA-1 on Jurkat T cells using single-particle tracking (SPT) methods (Saxton and Jacobson, 1997; Jaqaman et al., 2008; Alenghat and Golan, 2013). Cells were labeled with Cy5-conjugated anti-LFA-1 (clone TS2/4) at low enough concentrations to achieve sparse labeling of the receptors as observed by TIRF. Videos were recorded and analyzed to determine trajectories of LFA-1 on live cells (10 s, 10 FPS). This strategy allowed us to obtain many trajectories rapidly; however, due to photobleaching, trajectories recorded in this experiment tend to be shorter than those obtained from tracking of polystyrene beads or quantum dots. Trajectories were analyzed with u-Track and processed with custom scripts in MATlab (Cairo et al., 2006; Jaqaman et al., 2008). Data were pooled from multiple cells for each condition and are summarized in Table 1. All diffusion measurements were calculated as microdiffusion coefficients (D_micro_) due to the short duration of the trajectories (Qian et al., 1991). Our observations were in general agreement with SPT studies of fusion-protein labeled LFA-1 (Ishibashi et al., 2015). In previous SPT observations of LFA-1 at high time resolution the diffusion coefficients were found to have a non-normal distribution (Cairo et al., 2006). We found this to also be the case in our SPT data set, as the measured diffusion coefficients spanned up to four decades. Therefore, we proceeded to analyze these data as normal and lognormal distributions (Table S1 and Figure S1). Comparisons of the linear and logarithmic means found that LFA-1 on cytoD- and NEU3-treated cells exhibited significantly increased diffusion. Beanplots showing the distribution of diffusion coefficients are shown in Figure 5, and illustrate the shifts in LFA-1 diffusion in logarithmic scale (Kampstra, 2008). Further analysis of these data as cumulative distribution functions (CDF) illustrate the clear increase in LFA-1 diffusion upon NEU3 treatment. Our measurements were in general agreement with previous studies of LFA-1 lateral mobility using other methods (Gaborski et al., 2013). These data allowed us to conclude that NEU3 had a significant positive effect on the lateral mobility of LFA-1. Interestingly, the bacterial neuraminidase, NanI, had no significant effect on LFA-1 mobility in this experiment. These data support a specific role for NEU3 enzyme activity in the regulation of integrin mobility.

**Table 1:** Diffusion of LFA-1 determined using SPT.

**Condition**	***N***	**D**_****micro****_^^**†**^^			
		**Mean (linear)**	**Median (linear)**	**Mean (log transformed)**	**Median^‡^ (log normal)**
DMSO (control)	321	5.2 ± 0.3	3.28	2 ± 1	2.2 ± 0.4
PMA	334	5.9 ± 0.4	4.31	2 ± 1	2.4 ± 0.5
cytoD	422	7.7 ± 0.7^**^	4.32	3 ± 1^**^	3.0 ± 0.5
Buffer (control)	294	6.1 ± 0.6	3.28	2 ± 1	1.8 ± 0.4
NEU3	210	11 ± 1^****^	6.11	4 ± 1^****^	4.3 ± 0.9
NanI	216	5.5 ± 0.4	3.32	2 ± 1	2.3 ± 0.5

*Data was analyzed using u-Track (Jaqaman et al., 2008) and custom scripts implemented in Matlab (Cairo et al., 2006). Values listed are either the arithmetic mean, arithmetic median, or the median determined for a log normal distribution (see Supporting Information). Error is given as the standard error of the mean*.

† *Diffusion coefficients are in units of × 10^−10^ [cm^2^s^−1^] or × 10^−2^ [μm^2^s^−1^]; Data were compared to the appropriate control using a t-test to determine p-values*;

** *p ≤ 0.01*;

**** *p ≤ 0.0001*.

‡ *Median calculated based on a lognormal fit as described in Supporting Information*.

### LFA-1–ICAM-1 Adhesion Was Blocked by NEU3

**Figure 6:**
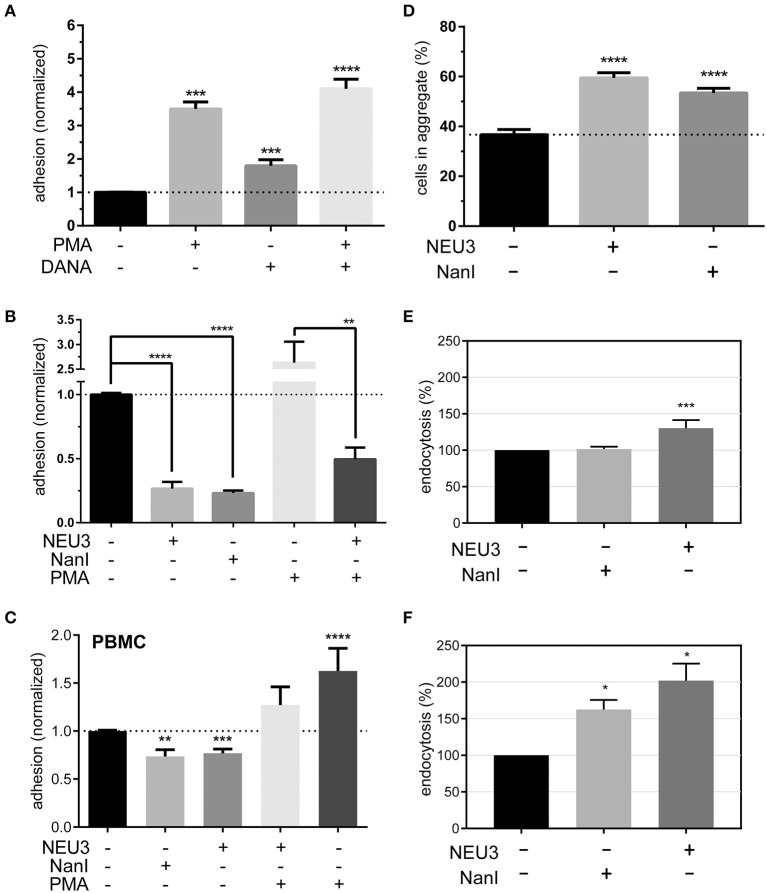
Adhesion of T cells is altered by Neu treatment. **(A)** Adhesion of Jurkat cells to ICAM-1 was determined using flow cytometry and fluorescent beads (1 μm) under the indicated conditions. Control samples were treated with DMSO (0.05 %), PMA, or DANA for 30 min. **(B)** Adhesion of Jurkat cells to ICAM-1 was determined using flow cytometry and fluorescent beads; samples were treated with buffer or enzyme for 3 h, followed by incubation with buffer, PMA, or DANA (100 μM) for 30 min. All cytometry data were normalized to the appropriate control after background subtraction (BSA coated beads). **(C)** Isolated PBMC were treated as indicated and adhesion to ICAM-1 coated beads was determined by flow cytometry. Data shown are from at least three healthy donors for each condition. Values shown in **(A–C)** are the average of *N* = 6–12 replicates, error is the standard error of the mean. **(D)** Homotypic aggregation of Jurkat cells was determined using microscopy. Cells were incubated under the indicated conditions for 3 h. Aggregation was determined by imaging to determine the total number of cells and the number of cells found within aggregates. Aggregation is expressed as the percentage of cells in all samples found within an aggregate (*N* = 24, from two separate experiments), and error is shown as the standard error of the mean. **(E)** Changes in the endocytosis of β2-integrin (*N* = 4) and **(F)** β1-integrin (*N* = 2) in Jurkat cells after treatment with NanI or NEU3 (30 min at 37°C). Error bars are shown as standard error of the mean, all data were compared to the indicated control using a *t*-test; *p ≤ 0.05; **p ≤ 0.01; ***p ≤ 0.005; ****p ≤ 0.0001.

To determine if NEU3 had a functional effect on LFA-1–mediated adhesion we employed a flow cytometry-based assay, similar to previous reports (See Experimental Procedures section) (Crucian et al., 2006). Fluorescent polystyrene beads were coated with recombinant ICAM-1, and cell–bead conjugates were detected by flow cytometry. Binding to beads was normalized to ICAM-1 coated beads as a positive control and bovine serum albumin (BSA) coated beads as a negative control. We found that PMA treatment of Jurkat cells increased adhesion as expected (Figure 6A). To test the role of native neuraminidase enzymes, we treated the cells with a general neuraminidase inhibitor, 2,3-dehydro-2-deoxy-*N*-acetylneuraminic acid (DANA). DANA is known to inhibit multiple human NEU isoenzymes (Cairo, 2014; Richards et al., 2018), and we observed a significant increase in LFA-1 adhesion after DANA treatment in the absence and presence of PMA. This result was consistent with a role for native NEU activity that negatively regulates LFA-1–ICAM-1 adhesion. Treatment of cells with purified NEU3 or NanI resulted in a dramatic block of LFA-1–ICAM-1 adhesion (Figure 6B). Experiments with a NEU3(Y370F) mutant confirmed that the effect of NEU3 was due to its enzymatic activity (Figure S2) (Albohy et al., 2010). Furthermore, to resolve the likely substrate of each enzyme we performed additional controls. Control experiments with ICAM-1 beads pre-treated with either NEU3 or NanI found that NEU3 treatment had no effect on ICAM-1–LFA-1 adhesion; while treatment of the same beads with NanI resulted in a large decrease in adhesion. Thus, we concluded that the two enzymes inhibited adhesion through modification of different substrates: Treatment with NanI blocked adhesion as a result of desialylation of the ICAM-1 ligand, while NEU3 blocked adhesion through enzymatic modification of a cell-surface target.

We further confirmed our observations beyond model cells by testing the effect NEU3 on ICAM-1 adhesion in PBMC (Figure 6C). Treatment of PBMC with NEU3 or NanI enzymes resulted in a significant decrease in ICAM-1 adhesion. Notably, NEU3 treatment of Jurkat and PBMC cells partially suppressed PMA-activated adhesion suggesting a regulatory role for the enzyme late in the activation pathway.

### NEU3 Enhanced Homotypic Aggregation

Once we had concluded that NEU3 could act as a negative regulator of β2-integrin mediated adhesion, we investigated the effect of NEU3 on an alternative cell adhesion process—homotypic aggregation. The homotypic aggregation of T cells is generally considered to be mediated by multiple receptors (Kansas and Tedder, 1991; Andrew et al., 1994; Cho et al., 2001) including LFA-1 (Rothlein and Springer, 1986) and VLA-4 (Bednarczyk and McIntyre, 1990; Campanero et al., 1990). We determined the number of cells involved in aggregates using microscopy (Figure 6D and Figure S3). Cells were treated with NEU3 or NanI, both of which resulted in significantly increased aggregation. Previous results have found that NEU3 increased fibronectin–β1 integrin cell migration in epithelial cells, and the effect was not due to desialylation of fibronectin (Jia et al., 2016). Homotypic aggregation of neutrophils has been reported to be increased by treatment with NanI (Cross and Wright, 1991). We concluded that while NEU3 disrupted LFA-1–ICAM-1 interactions (*vide infra*), desialylation of cell surface targets by NEU3 or NanI could also stimulate other adhesion mechanisms. These two results indicate that sialic acid can be either activating or inhibitory in adhesion, likely due to the specific target SGC involved.

### NEU3 Altered Endocytosis of **β**1 and **β**2 Integrins

Previous studies have supported a role for glycolipids in the regulation of integrin endocytosis (Sharma et al., 2005). Our examination of the effect of NEU3 on integrin adhesion suggested differential regulation of these two adhesion receptors. Perturbation of the balance of exo- and endocytosis of integrins is well-known as a mechanism to regulate adhesion (Caswell and Norman, 2006; Pellinen and Ivaska, 2006). We used biotin labeling of cell-surface proteins to interrogate changes in endocytosis of β2 and β1 integrins in Jurkat after exposure to NEU enzymes. We observed a significant increase in endocytosis of the β2 integrin after NEU3 treatment, but NanI appeared to have no significant effect (Figure 6E). In contrast, the β1 integrin showed a large increase in endocytosis after both NEU3 and NanI treatment (Figure 6F). We note that these analyses are based on densitometry of multiple western blots and are best interpreted qualitatively.

### Neuraminidases Altered Expression of LFA-1 Epitopes

**Figure 7:**
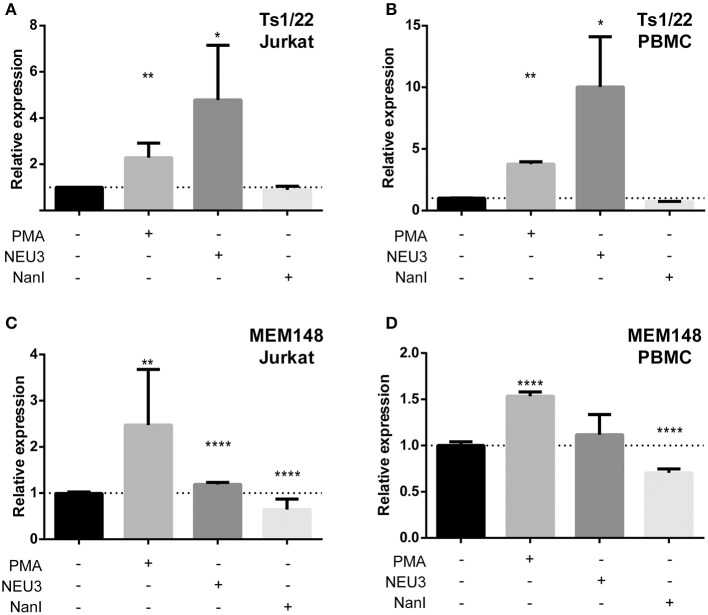
Alteration of LFA-1 epitopes by neuraminidase treatment. Jurkat T cells **(A,C)** or PBMC **(B,D)** were treated with the indicated conditions. Treated cells were labeled with primary antibodies (TS1/22 or MEM148), followed by an AF647-conjugated secondary antibody. Cells were fixed with 1% PFA, analyzed by flow cytometry, and normalized to control (buffer) treatment. Data shown are the mean of three replicates for each sample and error is shown as the standard error of mean. Data were compared to the appropriate control using a *t*-test to determine *p*-values; *p ≤ 0.05; **p ≤ 0.01; ****p ≤ 0.0001.

Our observation that NEU3 activity blocked LFA-1 adhesion could be the result of multiple mechanisms. To gain some insight into the process, we measured changes in known surface epitopes of LFA-1. The MEM148 epitope is found in the membrane proximal domain of CD18, and is an activation-dependent epitope of LFA-1 (Drbal et al., 2001). The TS1/22 antibody binds to the LFA-1 α-chain, and is both adhesion blocking and conformationally independent (Kuwano et al., 2010). To detect changes in epitope expression after treatment with NEU, we used flow cytometry (Figure 7). The TS1/22 epitope showed a large increase in expression on both cell types after NEU3 treatment, while NanI had no detectable effect on this epitope. Treatment of cells with NEU3 showed a significant increase in the MEM148 activation epitope on Jurkat, but not on PBMC. NanI treatment resulted in a decrease in the MEM148 epitope on both cell types. These data are consistent with increased LFA-1 total expression levels upon NEU3 treatment, with minor changes to the MEM148 activation epitope. Increased LFA-1 expression may be a result of delivery of LFA-1 to the surface from intracellular stores (Miller et al., 1987). We note that previous reports have observed increased expression of the MEM148 epitope and increased surface-localized LFA-1 on neutrophils after NanI treatment (Feng et al., 2011).

## Discussion

Our data demonstrated that the human NEU3 enzyme can act as a negative regulator of LFA-1–ICAM-1 mediated adhesion in lymphocytes. We confirmed that treatment of cells with exogenous NEU3 produced a reduction of sialylated glycolipids (e.g., GM3) on cells and reduced sialylation of the LFA-1 glycoprotein *in vitro*. NEU3 has been previously shown to prefer glycolipid substrates over glycoproteins, due to its requirement for substrates with a hydrophobic aglycone (Ha et al., 2004; Sandbhor et al., 2011). Treatment of cells with NEU3 or NanI (which lacks activity for gangliosides) (Peter et al., 1995), produced increased LFA-1 clustering as observed by fluorescence microscopy. Quantitative analysis of single-cell images confirmed that LFA-1 clustering was increased in NEU-treated cells. Analysis of the lateral mobility of LFA-1 found that NEU3 treatment resulted in an increase in integrin diffusion. Importantly, substantial effects on lateral mobility were only observed after NEU3 treatment, consistent with a role for gangliosides in regulating integrin diffusion (Sharma et al., 2005). NEU activity had a significant influence on LFA-1–ICAM-1 adhesion, and we concluded that NEU3 blocked LFA-1 adhesion through desialylation of a cell-surface target (e.g., glycolipids or glycoproteins). Control experiments confirmed that the NEU3 anti-adhesive effect was not a result of changes to ICAM-1 glycosylation, as was the case for NanI. Furthermore, we confirmed that NEU3 activity was distinct from NanI in that NEU3 induced an increase in surface expression of LFA-1. We observed that NEU3 activity increased endocytosis of both β1 and β2 integrins while NanI only affected the β1 integrin, suggesting a role for glycolipids in regulating the balance of exo- and endocytosis of adhesion receptors. We note that our experiments have focused on exogenous NEU3, which is a limitation of our study. However, the observation that treatment with an inhibitor of native NEU activity (DANA) blocks LFA-1-mediated adhesion is consistent with a role for native NEU. Together, our results implicate NEU3 as a potential regulator of β2-integrin mediated adhesion.

LFA-1 activity is governed by the interplay of avidity and affinity regulation; and these factors correspond to receptor clustering and conformational change, respectively (van Kooyk and Figdor, 2000; Carman and Springer, 2003; Kim et al., 2004). A quantitative analysis of single-cell images obtained by TIRF microscopy confirmed that a larger proportion of LFA-1 was found in clusters on NEU–treated cells. In accord with this observation, we found that the lateral mobility of LFA-1 on NEU–treated cells was increased, providing a mechanism for the change in integrin organization (Cairo and Golan, 2008). LFA-1 has been observed to cluster in microdomains (Scheiermann et al., 2010) with tightly regulated lateral mobility (Bakker et al., 2012). The size of LFA-1 clusters has been estimated over a wide range between 50 and 200 nm (Cambi et al., 2006; van Zanten et al., 2009; Rajani et al., 2011). Our observations using TIRF microscopy are limited by diffraction, and therefore changes observed for cluster size (Figure 4) were likely due to co-localization of multiple microclusters. The lateral mobility of LFA-1 has been found to be dependent on conformational state and stimulation of the cell (Cairo et al., 2006; Alenghat and Golan, 2013). Furthermore, LFA-1 mobility is linked to the ability of the cell to form a stable adhesion (Ishibashi et al., 2015).

The two NEU enzymes studied here had distinct effects on LFA-1. We observed that NEU3 activity induced an LFA-1 activation epitope on Jurkat, but had no effect on PBMC (Drbal et al., 2001). Treatment of cells with NanI showed a uniform decrease in the MEM148 activation epitope. Glycosylation of integrins is known to influence both conformation and function (Bellis, 2004; Gu and Taniguchi, 2004; Liu et al., 2008), and our lectin blots confirm that these enzymes modify glycosylation of LFA-1. While NanI activity also resulted in a blockade of LFA-1–ICAM-1 interactions, this effect can be ascribed to modification of the ICAM-1 ligand, rather than targets on the lymphocyte. These observations may be specific to cell type, as NEU activity directed at LFA-1 is reported to enhance adhesion of neutrophils (Feng et al., 2011). In addition to conformational changes of integrins, desialylation may alter inter-molecular interactions which depend on the revealed galactoside epitopes generated by NEU activity (Zhuo et al., 2008; Yang et al., 2017).

Signaling mechanisms known to negatively regulate LFA-1 include the protein tyrosine phosphatase receptor type γ (Mirenda et al., 2015), and the Lyn kinase (Nakata et al., 2006; Malik et al., 2008). Notably, Lyn activity suppresses LFA-1–ICAM-1 adhesion, but enhances cell migration. Lyn activity is known to be regulated by glycolipid composition of the outer leaflet (Sonnino et al., 2010). Furthermore, Lyn is known to be found at the leading edge of migrating cells (He et al., 2011), as is NEU3 and consistent with our imaging in Figure 3 (Yamaguchi et al., 2006). We note that Lyn-mediated activation of alternative adhesion mechanisms is consistent with our homotypic aggregation results (Figure 6). Future investigations will need to address a link between Lyn and NEU3 as it pertains to LFA-1 down-regulation. While our data suggest NEU3 is a negative regulator of LFA-1 adhesion, we also observed an increase in homotypic aggregation in NEU3-treated cells. Homotypic aggregation of lymphocytes is mediated by receptors including the αLβ2 (Rothlein and Springer, 1986), α4β1, and α5β1 integrins (Caixia et al., 1991). The activity of NEU3 on simple gangliosides (e.g., GM3) would generate neutral glycosphingolipids, which are known to be activators of homotypic aggregation in hematopoietic cells (Yamaji et al., 1997). Furthermore, changes in membrane cholesterol or GM1 are known to disrupt LFA-1 adhesion, and our results may suggest that changes to other glycolipid components of microdomains have a similar effect (Marwali et al., 2003). Increased NEU3 activity may alter the concentration of additional degradation products of GSL. Ceramide is known to increase surface expression of β2 integrin, and to block β2-integrin–dependent adhesion while preserving homotypic aggregation (Feldhaus et al., 2002).

How do NEU enzymes affect LFA-1 function? First, the mechanisms of action of each NEU enzyme used in this study have important differences. The substrate profile of each enzyme is different, with NanI acting on glycoproteins while NEU3 uses glycolipids as its favored substrates (Peter et al., 1995; Wang Y. et al., 2001). Thus, NanI treatment is likely to alter glycoprotein substrates, while NEU3 modifies both glycolipids and LFA-1. Changes to glycoprotein epitopes of LFA-1 may block adhesive interactions (Ardman et al., 1992) or induce engagement of new protein-glycan interactions (Wang X.Q. et al., 2002; Rossi et al., 2006; Rabinovich et al., 2007). Galectins are secreted lectins that bind to β-galactoside epitopes, which are often revealed by NEU activity (Rabinovich and Toscano, 2009). Galectin-1 can inhibit leukocyte adhesion (He and Baum, 2006; Norling et al., 2008), while Galectin-3 can promote neutrophil adhesion (Kuwabara and Liu, 1996). Previous work has found that native NEU activity in neutrophils positively regulates LFA-1 adhesion, which may implicate other isoenzymes, such as NEU1, for this activity (Feng et al., 2011). NEU3 activity altered lipid composition and could therefore influence membrane microdomain recruitment, function, or trafficking of integrins (Gopalakrishna et al., 2004; Ledeen and Wu, 2007; Nakayama et al., 2008). It is possible that direct integrin-glycolipid interactions are responsible for the reorganization of LFA-1 observed here, as β1-integrins are known to bind directly to gangliosides (Wang X. et al., 2001). Indeed, changes in membrane glycolipid composition have been shown to affect the recruitment of integrins and Src kinases to membrane microdomains (Kazui et al., 2000). Ectoenzymes are emerging as important regulators of leukocyte migration (Salmi and Jalkanen, 2005). NEU3 has been established as a plasma membrane-associated enzyme (Zanchetti et al., 2007), and our work here confirms that its activity can negatively regulate leukocyte adhesion.

The data presented here provide evidence that the human NEU3 enzyme can act as a negative regulator of LFA-1–ICAM-1 adhesion. However, it is important to emphasize that the enzyme also activates other cell-adhesion mechanisms (Figure 6D). The enzyme alters glycolipid composition, which likely leads to a shift in clustering and increased endocytosis of LFA-1. This mechanism of LFA-1 regulation was able to substantially block PMA-activated adhesion of leukocytes, and may present a novel target for pharmacological intervention in inflammation (Cairo, 2014). Although blocking of NEU3 would result in positive regulation of LFA-1, inhibition may also block the downstream adhesion mechanisms which are activated by NEU3. Future work should address the specific adhesion mechanisms which NEU3 may positively regulate, and the role of NEU3 within the inflammatory cascade (Ley et al., 2007).

## Experimental Procedures

### Cell Culture

Jurkat cells (clone E6.1) were grown in RPMI 1640 medium with 10% fetal bovine serum (FBS) at 37°C under 5% CO_2_ to ~1.5 × 10^6^ cells mL^−1^. Phorbol 12-myristate 13-acetate (PMA) (Sigma-Aldrich. Oakville, Ontario, Canada) and cytochalasin D (cytoD) (ENZO Life Sciences. Farmingdale, NY, USA) were dissolved in dimethyl sulfoxide (DMSO) as stock solutions (Sigma-Aldrich. Oakville, Ontario, Canada). Human neuraminidase 3 (NEU3) and NanI (Sigma-Aldrich. Oakville, Ontario, Canada) were stored in the same NEU3 buffer (0.2 M NaCl, 10% glycerol, 10 mM maltose, 20 mM MOPS pH 7.2). NEU3 was produced as a recombinant MBP fusion as previously reported (Albohy et al., 2010).

Peripheral blood mononuclear cells (PBMC) were isolated from whole blood samples of healthy donors following a protocol approved by the Health Research Ethics Board of the University of Alberta (Pro00016491). Briefly, cells were centrifuged over a ficoll gradient, transferred to a culture flask, and incubated overnight at 37°C with 5% CO_2_ in RPMI medium (10% FBS and 1% penicillin-streptomycin).

Cell treatments were performed using the conditions indicated below. A suspension of 1 × 10^6^ cells was taken from the culture flask and washed three times with buffer. In all washing steps, the cells were spun at 1,200 rpm for 2 min in a desktop centrifuge. For PMA and cytoD conditions, cells were re-suspended in 1 mL of buffer (PBS) with DMSO (0.05% final concentration) or the same buffer with DMSO containing PMA (200 ng mL^−1^) or cytoD (2.5 μg mL^−1^). The samples were then incubated at 37°C under 5% CO_2_ for 30 min. For enzyme treatments, cells were re-suspended in PBS alone, NEU3 enzyme (0.01875 U), or NanI enzyme (0.01875 U). One unit of enzyme activity was defined as, 1 U = 1 μmol 4MU-NANA substrate cleavage min^−1^, this calibration was done at pH 4.5. Enzyme samples were then incubated at 37°C under 5% CO_2_ for 3 h. After incubation all treated cells were then washed 3 times with PBS before further labeling, analysis, or extraction steps.

### High-Performance Thin Layer Chromatography (HPTLC)

For high-performance thin layer chromatography (HPTLC) experiments, phosphate buffered saline (PBS) was used as washing buffer. All treatments were done with 1 × 10^7^ cells in a 10 mL volume. After treatment, the cells were centrifuged to a pellet and re-suspended in 60 μL water, and sonicated for 30 s. Cells were extracted with a mixture of chloroform and methanol (1:1, 400 μL × 3) and agitated for 10 min. The sample was centrifuged (10,000 rpm for 10 min), and the supernatant was transferred to a glass bottle, dried under a flow of N_2_, and stored at −20°C. Before analysis, the cell extract was dissolved in a chloroform and methanol solution (1:1, 200 μL) and applied to a HPTLC plate (Sigma-Aldrich) using a glass micropipette. Chromatography was performed with acetic acid, *n*-butanol, and 0.25% CaCl_2_ (1:2:1) as the eluent followed by staining with orcinol (0.5 g orcinol, 200 mL 8% H_2_SO_4_ in ethanol).

### Extraction and Purification of Gangliosides

Ganglioside extraction and purification was performed following previous reports (Neville et al., 2004). Briefly, a lysate of 1 × 10^6^ Jurkat T cells was diluted with ice cold water (4 mL g^−1^ based on weight of sample). After homogenization, methanol was added to make the final ratio of methanol:water 8:3. Chloroform was added after vigorous mixing of this suspension to make the chloroform:methanol:water mixture to the ratio 4:8:3 (v/v/v). This mixture was vortexed and centrifuged at 1,500 RPM for 15 min. The supernatant was carefully recovered, volume measured, then diluted with 0.173 volumes of water. After mixing, the suspension was centrifuged again. The upper phase was recovered and transferred to a fresh tube, purified on a SepPak C18 cartridge (Waters Corporation, Milford, MA, USA), evaporated to dryness under a stream of nitrogen, and re-dissolved in methanol.

### LC-MS Analysis of Gangliosides

Expression and purification of EGCase was performed following previous reports with a pET30 vector (Albrecht et al., 2016). Samples of extracted GSLs were dissolved in a 50 mM sodium acetate buffer (pH 5.2) containing 1 mg mL^−1^ sodium cholate and incubated for 18 h at 30°C with 0.086U EGCase. One unit of EGCase I was defined as the amount of enzyme that hydrolyzes 1 μmol of GM3 per minute at 30°C. Released glycans were labeled with a mixture containing 30 mg anthranilic acid, 20 mg boric acid, 40 mg sodium acetate, and 45 mg sodium cyanoborohydride at 80°C for 45 min.

Labeled glycans were analyzed using an Agilent 1200 SL HPLC system and a normal-phase column (Accucore-150-Amide-HILIC, 2.6 μm, 2.1 × 150 mm, Thermo Fisher). Dried samples were re-solubilized in water:DMF:acetonitrile in the ratio 1:1:2 and 15 μL was injected. The fluorescence detector was set to monitor at 320 nm excitation and 420 nm emission, and all chromatography was performed at 40°C. Mass spectra were acquired in negative mode using an Agilent 6220 Accurate-Mass TOF HPLC/MS system with a dual spray electrospray ionization source along with a secondary reference sprayer for a reference mass solution. Data analysis was performed using the Agilent MassHunter Qualitative Analysis software package version B.07.01.

### Single-Particle Tracking of Integrin Receptors

In single particle tracking and TIRF imaging experiments the washing buffer was HBSSB (1% BSA, Hank's Balanced Salt Solution). TS2/4 mAb was purified from HB244 cell line (American Type Culture Collection, ATCC). A Cy5-antibody conjugate was generated using an NHS ester of Cy5 (GE Healthcare, Buckinghamshire, UK) using the manufacturer's protocol. The dye:antibody ratio was measured at 3.7 dye per antibody after purification. A final concentration of 30 ng mL^−1^ of labeled protein was added into a sample of 1 × 10^6^ treated cells. Cells were washed 3 times with HBSSB buffer after labeling at 37°C for 15 min. Labeled cells were re-suspended in 1 mL HBSSB and transferred to a 24-well cell culture plate containing a coverglass which was previously treated with 10 μg mL^−1^ of poly-L-lysine. The plate was spun at 400 g for 7 min, and the well was washed 3 times with HBSSB to remove unattached cells. The coverglass was transferred onto a microscopy slide and sealed with Cytoseal 60 (Thermo Fisher Scientific, Waltham, MA). All tracking data were acquired within 30 min of sealing. Tracking videos were taken on a NIKON Ti TIRF microscopy with 638 nm laser excitation and a 690 ± 40 nm filter with a 60 × TIRF objective (NA 1.49) with an additional 1.5 × magnifier (providing a final resolution of 252 nm pixel^−1^). Videos were acquired at 10 FPS for 10 s and analyzed with u-track (Jaqaman et al., 2008) in Matlab (2012b). Trajectories with fewer than 20 steps were discarded. The intensity of the trajectories was used to exclude the top and bottom 5 % of trajectories from the analysis. The data were processed using custom scripts in MATlab (Cairo et al., 2006).

### Total Internal Reflection Fluorescence Microscope (TIRF) Imaging and Cluster Analysis

Cells were treated identically to those used for SPT, followed by fixation with 1% paraformaldehyde (PFA) in PBS at 4°C for 60 min. The fixed cells were washed with PBS twice, and 2 × 10^5^ fixed cells from each treatment were re-suspended in 200 μL PBS. The cells were labeled with Cholera toxin B FITC (CTB-FITC, 5 μg mL^−1^; Sigma-Aldrich, Oakville, Ontario, Canada) and TS2/4-Cy5 (500 ng mL^−1^) at room temperature for 10 min. Labeled cells were washed twice with PBS, re-suspended in 1 mL PBS, and transferred to a 24-well cell culture plate containing a coverglass (poly-L-Lysine treated). The plate was spun at 400 g for 7 min, and the well was washed 3 times with PBS. The coverglass was transferred to a microscope slide and sealed with Cytoseal 60, followed by imaging using TIRF. More than three independent labeling samples were imaged for each treatment.

TIRF imaging for cluster analysis was performed using an identical protocol as described above, with a lower concentration of the TS2/4-Cy5 conjugate (80 ng mL^−1^). Fifteen cells were chosen for analysis based on DIC (the cells were apparently healthy and round) and staining (TIRF image showed TS2/4 labeling on the whole cell). Images of individual cells were processed in ImageJ by applying a threshold to identify labeled pixels and processed using the analyze particle function to measure clusters larger than 4 pixel^2^ (0.07 mm^2^).

### ICAM-1 Adhesion Assay

To prepare ICAM-1–bead complexes purified ICAM-1 protein (5 μg, R&D systems, Minneapolis, MN, USA) was incubated with 25 μL of a 2% solution of 1 μm microbeads (yellow-green sulfate microspheres; Life Technologies, Burlington, ON, Canada) in a final volume of 100 μL (in 50 mM PBS, pH 8.3) for 8 h at 4°C. After incubation, a solution of PBS (50 mM) containing 2% BSA (100 μL) was added and the suspension of beads and was incubated overnight at 4°C. The beads were stored at 4°C and used within 24 h.

Jurkat T cells or PBMC were treated as above with PBS as the washing buffer. Treatment with DANA was at 100 μM. Treated cell samples contained 3 × 10^6^ cells in 1 mL PBS, and were labeled with 10 μL of ICAM-1 or control beads followed by incubation at 37°C for 15 min. The labeled cells were washed with PBS three times and resuspended in 1 mL of PBS followed by analysis on an Accuri C6 flow cytometer.

### Homotypic Aggregation of Jurkat Cells

Jurkat cells were washed with PBS three times and re-suspended in PBS at a concentration of 2 × 10^5^ cells mL^−1^. Cells were transferred to solutions with the following conditions: buffer alone, NEU3 (0.01875 U), or NanI (0.01875 U). All samples had a final concentration of 10% NEU3 storage buffer and 0.6% binding buffer (100 mM CAPS, 0.15M NaCl, 1 mM calcium chloride, pH 11.0). All samples were stained with 1 μg mL^−1^ Calcein AM (Life Technologies, Burlington, ON, Canada). Samples were transferred to a 96-well- plate (200 μL per well). The plate was incubated at 37°C for 3 h. Fluorescent images were taken with a NIKON Ti microscope using a 20x objective and a FITC filter set. Four images were taken for each well to provide 24 images for each condition. The images were analyzed with CellProfiler (Version 2.1.1) (Carpenter et al., 2006; Bray et al., 2015). The total number of the cells in each image, and the number of single cells (cells not in any clusters) were counted in CellProfiler. The amount of aggregation was calculated as (total number of cells – numbers of single cells)/total number of cells. Results were confirmed using at least two independent repeats.

### Integrin Endocytosis

Samples of sulfo-NHS-SS-Biotin and streptavidin-resin were obtained from Thermofisher, USA. Glutathione (GSH) was purchased from Sigma-Aldrich. Antibodies for β1 integrin (clone EP1041Y), β2 integrin (clone EP1286Y), HRP-conjugated goat anti-rabbit secondary antibody (ab6721) were obtained from Abcam, USA.

Biotin-based endocytosis assays were performed as previously described with slight modifications (Cihil and Swiatecka-Urban, 2013). Jurkat T cells were grown in 10% FBS-containing medium T-75 flasks (Corning, USA). Cells were collected by centrifugation at 300 g for 2 min and 2 × 10^6^ cells were placed in separate Eppendorf tubes. Samples were placed on ice and washed once with cold PBS, and then labeled with 0.8 mg mL^−1^ of sulfo-NHS-SS-biotin for 60 min at 4°C. Cells were then centrifuged again and unbound biotin was washed away with cold medium. Cells were then resuspended in prewarmed medium with or without treatment and biotin-labeled surface proteins were allowed to internalize at 37°C for 30 min. Enzyme treatments were performed in PBS buffer (pH 7.0) with 0.02 U of NEU3 or NanI. Cold medium was immediately added, and samples were put over ice. Any remaining biotin at the cell surface was removed with GSH buffer (75 mM sodium chloride, 1 mM magnesium chloride, 0.1 mM calcium chloride, 50 mM GSH, and 80 mM sodium hydroxide) for 30 min at 4°C, followed by multiple washes with cold PBS. The cells were pelleted and treated with lysis buffer [150 mM sodium chloride, 1.0% Triton X-100, 0.5% sodium deoxycholate, 0.1% SDS, 50 mM Tris, pH 8.0 and phosphatase and protease inhibitor cocktails (Roche, USA)] at 4°C for 30 min. The lysate was clarified by ultra-centrifugation at 18,000 × g for 10 min. Supernatants were collected, and a BCA assay was used to calibrate protein concentrations. Equal amounts of protein were incubated with streptavidin-resin with agitation at 4°C overnight. The resin was washed once with lysis buffer and boiled with 2x Laemmeli sample buffer containing 100 mM DTT. Endocytosed biotinylated β1 and β2 integrins were measured by separate western blots for the respective β-chains.

### LFA-1 Antibody Binding

MEM148 antibody was purchased from AbD Serotec (Raleigh, NC, USA); TS1/22 antibody was purchased from Fisher Scientific Ottawa, ON, Canada. A sample of cells (Jurkat or PBMC, 1 × 10^6^) were treated with NEU3 (0.01875 U) or NanI (0.01875 U) for 3 h, or PMA (200 ng mL^−1^) for 30 min. Incubations were done at 37°C at 5% CO_2_, followed by washing with PBS, centrifugation, and re-suspension in PBS buffer (900 μL). Cells were then divided into aliquots and labeled with TS1/22 or MEM148 antibodies for 30 min. Cells were then washed three times with PBS and re-suspended with PBS with AF-647–conjugated secondary antibody at 1:1000 dilution for 10 min. Cells were again washed three times and fixed with 1 % PFA for 10 min before analyzing using an Accuri C6 flow cytometer.

### Lectin Blotting of LFA-1

Purified LFA-1 (R&D systems, USA; 50 μg) was biotinylated with sulfo-NHS-SS-biotin and immobilized on Neutravidin resin (600 μL) overnight at pH 7. The suspension was then washed three times with PBS buffer (pH 7.0). The immobilized LFA-1 was then treated with NEU3 (0.01875 U) or NanI (0.01875 U) and the mixture was incubated for 3 h at 37°C. The resin was washed three times to remove contaminating proteins, followed by incubation at 95°C for 10 min in the presence of DTT (100 mM) to release LFA-1 from the resin. Buffer exchange was done to remove excess DTT, and a BCA assay was carried out to determine the protein concentration of each sample. Equal amounts of the protein were then loaded on an SDS-PAGE gel, transferred to a nitrocellulose membrane, and detected using biotinylated peanut agglutinin (PNA), *M. amurensis* agglutinin (MAA), or *S. nigra* (SNA) lectins (Bio-World, Ohio, USA) at 1:500 dilution. Lectins were imaged with streptavidin-HRP (1:200 dilution) and band intensity was analyzed using ImageJ.

## Data Availability Statement

All datasets generated for this study are included in the article/Supplementary Material.

## Ethics Statement

The studies involving human participants were reviewed and approved by University of Alberta, Health Research Ethics Board. The patients/participants provided their written informed consent to participate in this study.

## Author Contributions

MH designed and performed endocytosis, epitope expression, lectin blotting experiments, and wrote the manuscript. CL performed and analyzed fluorescence microscopy, SPT, antibody binding, FN aggregation experiments, and edited the manuscript. RC designed and performed glycolipid analysis and wrote the manuscript. CZ designed and performed ICAM binding experiments and produced NEU3 protein. NE designed antibody binding experiments. CC designed and coordinated the study, analyzed data, and wrote the manuscript.

### Conflict of Interest

The authors declare that the research was conducted in the absence of any commercial or financial relationships that could be construed as a potential conflict of interest.
