# Supplementary material for: Neuraminidase-3 Is a Negative Regulator of LFA-1 Adhesion
Source: Front Chem. 2019 Nov 22;7:791. doi: 10.3389/fchem.2019.00791 (PMC6882948; doi:10.3389/fchem.2019.00791)
Supplement: Supplementary file 1 [file Table_1.DOCX]

# Supporting information for:

# Neuraminidase-3 is a negative regulator of LFA-1 adhesion

Md. Amran Howlader, Caishun Li, Chunxia Zou, Radhika Chakraberty, Njuacha Ebesoh, and Christopher W. Cairo*

**Table of contents**

[Table S1: LFA-1 diffusion fit to a lognormal distribution 2](#_Toc23503701)

[Figure S1: Plots of Microdiffusion data from tracking of the β2 integrin. 3](#_Toc23503702)

[Table S2: LFA-1 Cluster Size by TIRF 4](#_Toc23503703)

[Figure S2: ICAM-1 adhesion controls. 5](#_Toc23503704)

[Figure S3: Homotypic aggregation controls. 6](#_Toc23503705)

[Figure S4: PNA lectin blot of LFA-1 treated with NEU3 and NanI. 7](#_Toc23503706)

[Figure S5: SNA lectin blot of LFA-1 treated with NEU3 and NanI. 8](#_Toc23503707)

[Figure S6: MAA blot of LFA-1 treated with NEU3 and NanI. 9](#_Toc23503708)

[Figure S7: LC-MS profiles of glycolipids. 10](#_Toc23503709)

[References 11](#_Toc23503710)

## Table S1: LFA-1 diffusion fit to a lognormal distribution

| Condition | n | mean‡  (log transformed) | p* | μ | σ | median†‡ |
| --- | --- | --- | --- | --- | --- | --- |
| DMSO | 321 | 2 ± 1 | - | -22.24 ± 0.08 | 1.61 ± 0.06 | 2.2 ± 0.4 |
| PMA | 334 | 2 ± 1 | 0.220 | -22.14 ± 0.10 | 1.77 ± 0.07 | 2.4 ± 0.5 |
| cytoD | 422 | 3 ± 1** | 0.005 | -21.93 ± 0.08 | 1.64 ± 0.06 | 3.0 ± 0.5 |
|  |  |  |  |  |  |  |
| Buffer | 294 | 2 ± 1 | - | -22.43 ± 0.11 | 1.88 ± 0.08 | 1.8 ± 0.4 |
| NEU3 | 210 | 4 ± 1**** | 1.08e-7 | -21.58 ± 0.11 | 1.63 ± 0.08 | 4.3 ± 0.9 |
| NanI | 216 | 2 ± 1 | 0.078 | -22.19 ± 0.11 | 1.62 ± 0.08 | 2.3 ± 0.5 |

‡Units are [x 10^-10^ cm^2^ sec^-1^].

*p value was calculated by transforming the data to log scale and comparing populations as two normal distributions. All samples were compared to control for significance.

†Median calculated as e^μ^. Error in the median was calculated as the difference between e^(μ+se)^ and e^(μ-se)^, where se is determined by the standard error of the fit (MATlab, dfittool).


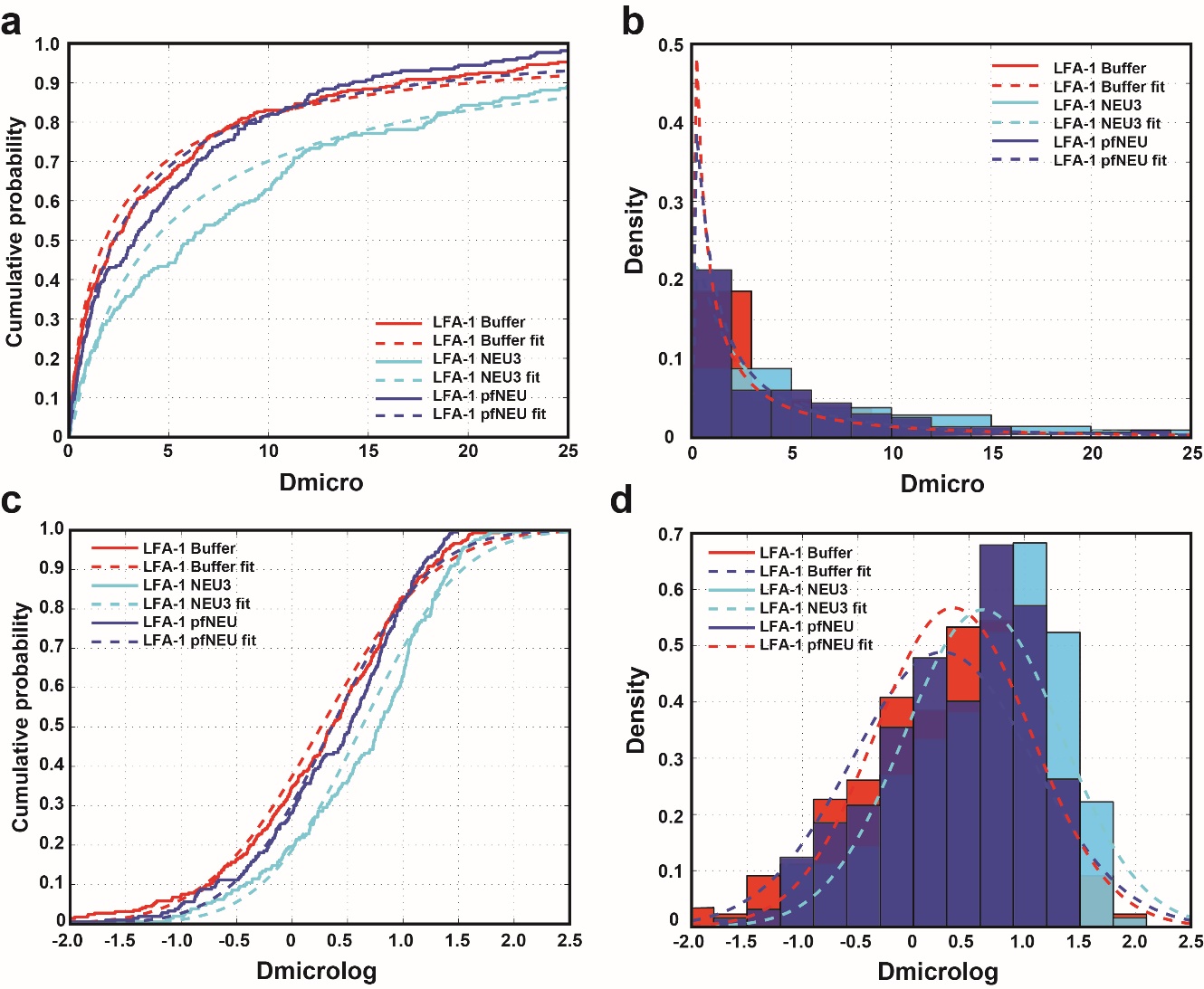


## Figure S1: Plots of Microdiffusion data from tracking of the β2 integrin.

(a) The empirical cumulative distribution function (CDF) was plotted and fit to a lnorm distribution. (b) A plot is shown of the population density function (PDF) with a lnorm fit overlayed. As an alternate way to present the data, the same data were subjected to a logarithmic transformation, and fit to a normal distribution. A fit of the normal distribution is plotted as the (c) CDF and (d) PDF. Distributions were plotted and fit using dfittool in MATlab, R2012. pfNEU refers to NanI treatment.

## Table S2: LFA-1 Cluster Size by TIRF

| Condition | n | Cluster size  [μm^2^ cell^-1^] | p | n | Cluster size†  [μm^2^ cluster^-1^] | p |
| --- | --- | --- | --- | --- | --- | --- |
| DMSO | 15 | 21.2 ± 1.2 | - | 126 | 2.0 ± 0.2 | - |
| PMA | 15 | 22.7 ± 1.2 | 0.4 | 122 | 2.4 ± 0.2 | 0.08 |
| cytoD | 15 | 25.6 ± 1.4 | 0.025 | 121 | 2.7 ± 0.3 | 0.015 |
|  |  |  |  |  |  |  |
| buffer | 15 | 16.7 ± 1.2 | - | 104 | 1.9 ± 0.2 | - |
| Neu3 | 15 | 22.4 ± 1.6 | 0.008 | 110 | 2.6 ± 0.3 | 0.08 |
| pfNeu | 15 | 35.7 ± 2.6 | < 0.0001 | 137 | 3.4 ± 0.4 | 0.004 |

*, p values were calculated using a t-test to the appropriate control (DMSO or buffer). †, Size of individual clusters was calculated after filtering out clusters smaller than 4 pixels^2^ (0.07 μm^2^). Error is given as the standard error of the mean.

## Figure S2: ICAM-1 adhesion controls.

Adhesion of Jurkat cells to ICAM-1 was determined using flow cytometry and fluorescent beads (1 μm) under the indicated conditions. Beads were coated with ICAM-1 for all experiments shown. Where indicated, the ICAM-labelled beads were pre-treated with the enzyme indicated in parenthesis (3 hrs pH 7.2), washed and then used for adhesion experiments. For ICAM+NanI, the beads were co-incubated with the enzyme during the adhesion experiment. Cells were treated with ICAM beads under conditions identical to Figure 6. The NEU3(Y370) mutant was prepared as previously reported.(*1*) Error bars are shown for SEM.

## Figure S3: Homotypic aggregation controls.

Homotypic aggregation of Jurkat cells was determined using microscopy. Cells were incubated under the indicated conditions for 3 hrs. Aggregation was determined by imaging and analysis with CellProfiler (version 2.1.1) to determine the total number of cells and the number of cells found within aggregates. Aggregation is expressed as the percentage of cells in all samples found within an aggregate (N = 24, from two separate experiments), and error is shown as the standard error of the mean. The NEU3(Y370) mutant was prepared as previously reported.(*1*)


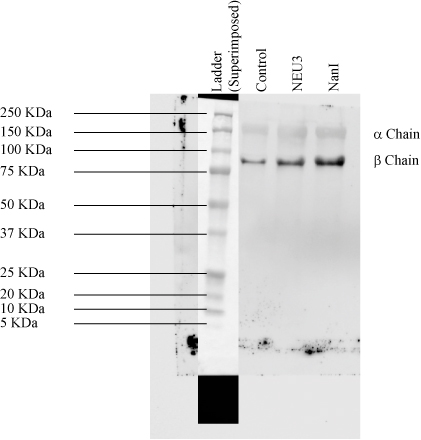


## Figure S4: PNA lectin blot of LFA-1 treated with NEU3 and NanI.

Purified LFA-1 was treated with NEU3 and NanI for 3 h at 37 °C. After thorough washing, LFA-1 was then blotted using biotinylated PNA lectin. Chemiluminescent blots were developed and analyzed for changes in band intensities. A representative run of two experiment are shown. Summarized analysis is shown in Fig 2c.


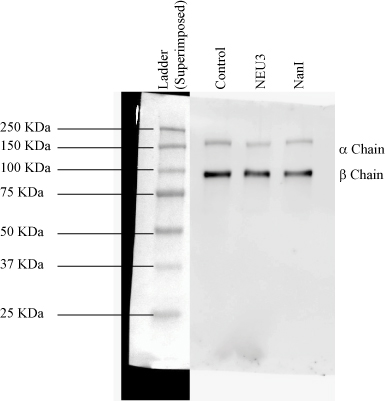


## Figure S5: SNA lectin blot of LFA-1 treated with NEU3 and NanI.

Purified LFA-1 was treated with NEU3 and NanI for 3 h at 37 °C. After thorough washing, LFA-1 was then blotted using biotinylated SNA lectin. Chemiluminescent blots were developed and analyzed for changes in band intensities. A representative run of two experiment are shown. Summarized analysis is shown in Fig 2b.


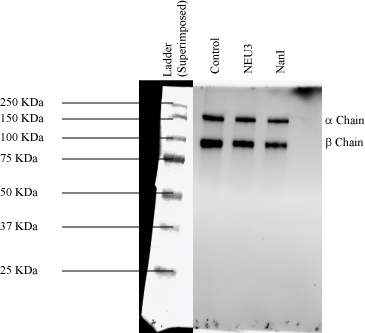


## Figure S6: MAA blot of LFA-1 treated with NEU3 and NanI.

Purified LFA-1 was treated with NEU3 and NanI for 3 h at 37 °C. After thorough washing, LFA-1 was then blotted using biotinylated MAA. Chemiluminescent blots were developed and analyzed for changes in band intensities. A representative run of two experiment are shown. Summarized analysis is shown in Fig 2c.

## Figure S7: LC-MS profiles of glycolipids.

Glycolipids were extracted from Jurkat cells under the indicated conditions and analyzed using LC-MS. Four replicates of each condition were performed, and one set of representative chromatograms are shown. See Figure 1b for quantitation.

## References

1. Albohy, A., Li, M. D., Zheng, R. B., Zou, C., and Cairo, C. W. (2010) Insight into substrate recognition and catalysis by the mammalian neuraminidase 3 (NEU3) through molecular modeling and site directed mutagenesis, *Glycobiology* *20*, 1127-1138.
